# Differences in self-reported health between low- and high-income groups in pre-retirement age and retirement age. A cohort study based on the European Social Survey

**DOI:** 10.1016/j.hpopen.2022.100070

**Published:** 2022-04-11

**Authors:** Jürgen Bauknecht, Sebastian Merkel

**Affiliations:** a Hochschule Koblenz, Koblenz, Germany; b Faculty of Social Science, Ruhr-University Bochum, Bochum, Germany

**Keywords:** Self-rated health, Health inequality, Aging, Europe

## Abstract

Using data from the European Social Survey 1 (2002) and 9 (2018) we show the development of self-rated health of older persons in 17 countries. We find a considerable increase of older persons reporting good or very good health between 2002 and 2018; this increase is similar in all groups examined. Absolute differences between income groups remained vastly stable. Further, in 2018 the high-income tercile of those between 65 and 80 years still reported better health than the low-income tercile of those between 49 and 64 years. Overall, self-rated health seems to have improved in Europe but there are still signs of a considerable gap between low-income groups and high-income groups.

## Highlights

• Older persons show significant increases in self-reported health from 2002 to 2018.
• Relative, but not absolute social inequality declined during this time.
• Self-reported health has increased with virtually every examined demographic.
• Gaps between high-income and low-income demographics remain significant, however.
• High-income, post-retirement groups report better health in 2018 than low-income, pre-retirement groups.

## 1 Introduction

Several studies have shown a correlation of income and health status within countries concluding that high/increasing income might have a positive effect on health [1], [2]. Moreover, health and income are interrelated, and multiple studies show that e.g. the transition into retirement may largely affect the (subjective) health status not only due to multiple physical, mental, and social aspects, but also due to decreased income [3], [4], [5], [6], [7]. At the same time, poor health might lead to early retirement.

In the nexus of health and income, older persons (aged 65 years and more) are a group of particular interest: On the one hand due to an increasing prevalence of chronic diseases and multimorbidity and on the other because of changing income situations e.g. due to retirement or death of a spouse. In view of the above-mentioned potentially positive effect of higher income on health the aim of our study is to analyse the differences in self-reported health between older persons in low- and high- income households, as well as the development of the health status within and between cohorts and the development of status-related health inequalities.

Therefore, our study depicts the state of self-rated health in older age cohorts in 17 countries in 2002 and 2018 using data of the European Social Survey (ESS). Cross-country comparisons bear additional challenges. Considering life expectancy Picket and Wilkinson argue that in an inter-country comparison there is “(…) not simply a lack of statistically significant relation with life expectancy, but no relation whatsoever. Average real incomes can be almost twice as high in some developed countries as in others without consequences for life expectancy“ [2]. Using gross national income per capita at purchasing power parities the authors conclude that “what matters may be social position, or income relative to others, rather than material living standards regardless of others” [2]. The European perspective allows us to show a general trend across countries in a comparatively long timespan. We focus on the association between income on health and analyse differences of self-rated health between the lowest and the highest income tercile. Our main hypotheses is that self-rated health of older persons in high-income households is significantly higher that of those living in low-income households. We want to investigate how differences have developed over time and between income groups. Therefore, these cohorts are analysed at two points in time and thus at a different age. By that, we answer the following research questions:

• Did the self-rated health status of older age cohorts between 49 and 64 years and between 65 and 80 years improve between 2002 and 2018?
• Did absolute inequalities between income groups increase between 2002 and 2018?

As the coverage of association between self-rated health and income is already extensive in the literature, the following section summarises selected findings focusing mainly on studies analysing self-reported health.

### 1.1 Health and income: A review of the literature

In a systematic narrative review, Read et al. [8] found and analysed 71 studies on health status of older (60 +) people in Europe published between 1995 and 2013. 44 of those studies covered self-rated health. Of those studies, 7 out of 7 analysing the association between self-rated adequacy of income and self-rated health show an association. However, the authors point out that “the association between income and self-rated health was less clear” [8], since out of 19 studies, only 11 reported such association.

An analysis of the European Values Study 2008/2009 shows that low income is significantly related to less-than-good-health in 23 of 42 European countries [9]. Based on ESS 8 (2016/2017) data from 23 countries, Papazoglou and Galariotis [10] confirm the assumption that higher individual income raises the probability of self-reported good or very good health.

Based on EU-SILC data, Forster et al. [11] show the differences in self-reported health between the lowest and the highest income quintile concerning the percentage reporting good health. Across the 28 EU countries at that time, the respective figures for both groups are 60% and 78% [11]. Further, they report on EQLS (European Quality of Life Surveys) data showing that between 2007 and 2016 the shares of people reporting bad health were stable in both, the bottom income quartile (14% and 13%) as well as the top quartile (around 5%).

Furthermore, an analysis of 42 European countries with data from the European Values Study (EVS) 2008/2009 shows higher income inequality being related to higher inequality in self-reported health [12]. Like the ESS, the EVS measures income via categories and not as an exact figure.

Overall, the highlighted studies show mixed results on the association between income and health status. This, however, is caused by different measures of income and health. Nevertheless, most studies listed show that there is a correlation between a high income and a good health status. Concerning long-term trends for past decades, several authors found increasing health inequalities (for European countries [13], [14]; for Central and Eastern European countries [15], [16]).

## 2 Methods

### 2.1 Dataset and timespan

The ESS is a bi-yearly cross-national survey in Europe, established in 2001. Data is collected via face-to-face interviews with newly selected cross-sectional samples. We chose ESS data (core questionnaire) because it covers a large period of time (2002 to 2018). As we use data from the ESS 1 (2002) and the ESS 9 (2018), we aim for the maximum time span coverable by the dataset. Two 8-year-steps (2002 to 2010 and 2010 to 2018) could have provided a distinction between rather recent developments and developments before 2010. Yet, we chose the 16-year step instead of two 8-year steps, since 2010 data may be affected by the 2008 financial crisis [9], [13], [16], [17], [18], [19], [20]. Our analysis contains all 17 countries which took part in both rounds. A complete list is shown in Table 1.

**Fig. 1:**
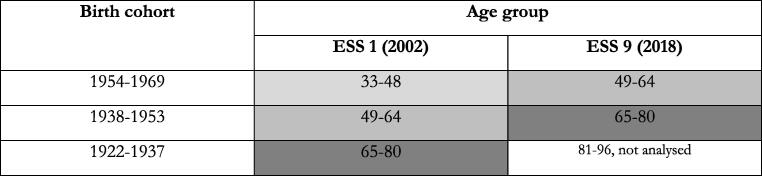
The ageing of the birth cohorts.

Although the ESS is no panel study, we created different age groups in a manner that birth cohorts enter the next higher age group between 2002 and 2018 (Fig. 1). We constructed the age boundaries based on the “classic” upper boundary for the “working age population” at age 64 used e.g. by OECD [21], although numerous European countries raised their legal retirement age beyond the age of 65.

The youngest birth cohort was middle aged (33–48 years) in 2002 and is roughly in pre-retirement age in 2018 (49–64 years). The second birth cohort (49–64 years in 2002) was in pre-retirement age in 2002 and in retirement age in 2018 (65–80 years). The oldest cohort (65–80 years) was in retirement age in 2002. This cohort is not analysed for 2018, since the numbers of cases between 81 and 90 years is too low (there are no persons older than 90 covered in ESS9 in the 17 countries). In sum, we can assess the development of the self-rated health status and health inequalities in this status for the youngest cohort (33–48 years in 2002) as they enter middle age (49–64 years) and for the second cohort (49–64 years in 2002) as they enter old age (65–80 years). The self-reported health status reflects an individual's perception of their social, biological, and psychological health. We can compare the self-rated health status and inequalities amongst those in old age (65–80 years) in 2002 and 2018.

### 2.2 Measure of health and income

As for the measurement of health, we use a single item. The item assesses the subjective perception of health (on a 5-point-scale: *very bad, bad, fair, good, very good*). Due to the ordinal scale of measurement, similar as other authors (e.g. [10], [22]) we dichotomised the variable into *very good* or *good* health on the one side and *fair, bad* or *very bad* health on the other side. This subjective assessment can be too positive or too negative, depending e.g. on individual psychological predispositions, lack of relevant information about one’s own health, and social desirability effects. Further, the question is about ‘health’. Only the footnote for further explanations by interviewers elaborates on the definition’s including ‘physical and mental health’. Therefore, it is possible that respondents answer this question mainly according to their physical health.

As we are interested in health differences between income groups in and between cohorts, it has to be noted how “different patterns of health inequalities emerge depending on the measure” [23]. Looking at the literature, studies either use income or education. For example, a study by Read et al. [8] has shown that education is used more frequently than income. Balaj et al. [24] analysed data from the ESS 7 (2014) rotating module on social inequalities in health and found considerable differences in the extent of health inequalities between the 21 countries [24]. They argue that education is less prone to reverse causation than income, which seems plausible given that the level of formal education is mainly determined in the first third of the life course.

Although ESS data also contains information on respondentś (and their spouseś) educational level (ISCED) we focus on income and argue that the position of a person in society is defined by income rather than formal education. Further, by using income adjusted for household composition, we focus on a gap in current research on health inequality between status groups. Using net household income adjusted for household composition according to the OECD equivalence scale [25], our study refines the methodology of previous studies on the relationship between income and health [10], [26], [27], [28], [29], [30]. It must be noted, however, that income is calculated in various ways across these studies. For example, Papazouglu and Galariotis [10] reduced the ten ESS categories of total net household income to five groups (deciles 1&2, 3&4, etc.). Jen at al. used income before taxes and not net income [26]. Mulatu and Schooler use family income not adjusted for household size [25]. Christelis et al. [28] used SHARE data and weight according to the OECD scale. Artazcoz et al. [29] measure household income and divide it by the square root of the number of people living in a household (with the square root calculation, couples living together, or families are estimated to be richer than with the OECD equivalence scales). Bovasso et al. [30] used 17 categories for personal and household income, yet did not mention any adjustment for household size.

According to their household’s net income adjusted to household composition, respondents are categorised into three terciles (low, medium and high income). We focus on the differences in self-rated health between the low and the high-income group.

As Kjellsson et al. [31] conclude, “it is generally a sensible idea to present the reader with both relative and absolute versions of inequality measures to compare inequality between populations”. Absolute inequality is often reported in comparisons of percentages of different groups in different times. Absolute health inequalities are defined as the gap in percentages of respondents in good or very good health between the low and the high-income tercile while relative inequalities are calculated as the percentual rise that would be needed to go from the lower value to the higher value. If 20 percent of the population in the low income tercile are in good or very good health and 40 per cent of those in the high tercile, the chances of a person in the high income tercile to be in good or very good health are twice as high as those of a person in the low income tercile. If later figures are at 40 and 60 percent respectively, the chances are only 50 per cent higher, so that absolute inequality remained stable, but relative inequality declined. Contrastingly, not percentages in good or very good health, but fair, bad or very bad health are measured, figures would be 80/60 in the beginning and 60/40 later, so that the differencesin the probabilities and therefore relative inequality would increased from 1,33 to 1,50. Therefore, although the concept of relative inequality seems to be plausible since it reflects individual probabilities, results on developments depend on an arbitrary choice.

## 3 Results & discussion

### 3.1 Development of self-rated health status

Across all four age groups, the share of respondents in good or very good health rose to a roughly similar degree (Table 1). For those aged between 49 and 64 in 2002 and 2018 amongst the low-income tercile, the share rose from 45.6 percent in 2002 to 53.9 percent in 2018. For the high-income tercile, the respective figures are 66.0 and 74.5 percent. Therefore, in 2018 the low-income tercile did not reach the value the high-income tercile already had in 2002. Amongst those in old age (65–80), in the low tercile the shares rose from 34.9 percent in 2002 to 41.8 percent in 2018, and for the high-income tercile from 51.4 to 59.7. This means that not only did the low-income tercile not reach the value in 2018 the high tercile already had in 2002, but also that the high-income tercile in 2018 amongst those between 65 and 80 had a higher value than the low-income tercile in the age group 49–64 (57.7 and 53.9 percent respectively). The high tercile’s value may be slightly inflated due to respondents comparing their health to others in the same age group (the opposite applying to the low tercile in the younger group). According to what is stated in the surveys however, those in old age of the high-income tercile feel healthier than the low-income tercile in the age group which is 16 years younger.

**Table 1:** Respondents in good or very good health (low and high-income tercile) in 17 European countries in 2002 and 2018 (percentage and numbers of cases).

		2002									2018					
		33–48			49–64			65–80			49–64			65–80		
		low	high	sig.	low	high	sig.	low	high	sig.	low	high	sig.	low	high	sig.
AT	%	86,0	87,9		47,4	52,6	*	42,3	63,2		62,6	75,4	**	51,6	60,1	
	n	166	153		91	101		22	24		129	156		63	89	
BE	%	74,4	92,0	***	63,1	77,0	*	64,4	57,3	*	61,1	78,6	***	60,8	76,1	*
	n	122	149		82	87		56	43		96	125		73	89	
CH	%	87,7	96,1	**	72,7	79,3		65,8	76,6		77,5	91,7	**	55,2	79,7	**
	n	193	173		104	111		50	72		93	100		37	51	
CZ	%	68,0	76,7		39,4	46,6		12,7	30,8	**	44,8	75,2	***	34,6	43,2	
	n	70	66		41	61		9	20		64	115		37	41	
DE	%	60,7	71,6	**	39,7	66,0	***	23,6	49,5	***	45,9	72,0	***	37,0	67,8	***
	n	165	189		93	128		25	54		101	157		60	97	
ES	%	71,9	78,9		46,7	67,5	**	22,7	38,7		53,2	59,5		40,4	46,0	
	n	69	75		35	54		15	24		59	66		23	29	
FI	%	72,6	83,4	*	40,1	75,0	***	28,9	51,2	**	54,7	77,3	***	38,4	63,8	***
	n	114	141		71	129,0		24	43		88	116		48	81	
FR	%	57,5	78,1	***	42,5	59,8	*	29,2	54,2	*	43,0	64,4	**	41,6	57,0	*
	n	77	107		45	58		14	32		71,0	114		47	65,0	
GB	%	72,3	86,7	***	57,0	76,6	***	45,7	70,2	***	47,3	83,9	***	54,0	79,1	***
	n	146	156		90	118		43	59		87	151		67	87	
HU	%	30,8	61,5	***	13,7	38,2	***	14,3	26,5		37,6	57,8	**	27,1	30,6	
	n	41	88		19	52		12	18		35	48		23	26	
IE	%	84,9	94,5	**	73,9	91,0	***	54,9	77,9	**	70,1	84,5	**	68,0	79,0	
	n	163	173		113	142		45	60		103	120		68,0	94,0	
NL	%	72,8	84,7	**	59,8	78,7	***	48,3	68,8	**	62,7	80,7	***	49,5	58,8	
	n	182	199		131	163		43	55		89	117		50	50	
NO	%	75,2	90,4	***	54,0	80,0	***	46,1	65,8	*	63,6	81,4	**	46,1	74,6	***
	n	167	198		101	136		41	48		77	96		35	53	
PL	%	50,9	65,1	*	17,5	45,5	***	12,8	27,9	*	45,7	72,0	***	13,7	35,0	**
	n	85	110		20	55		10	19		37	59		10	21	
PT	%	45,9	71,6	***	23,5	45,2	**	7,5	22,4	*	26,9	54,5	***	5,9	41,5	***
	n	45	68		19	33		4	17		21	42		3	22	
SE	%	74,7	86,5	**	56,7	77,9	***	53,5	55,1		78,6	86,1		58,1	76,2	**
	n	139	160		93	127		53	54		103	118		75	93	
SI	%	49,6	69,4	***	26,7	65,0	***	20,0	38,4	*	41,1	71,9	***	29,2	45,8	*
	n	67	84		28	67		12	28		46	87		21	38	
**MEAN**		**66,8**	**80,9**	***	**45,6**	**66,0**	***	**34,9**	**51,4**	***	**53,9**	**74,5**	***	**41,8**	**59,7**	***
**MEDIAN**		**72,3**	**83,4**	***	**46,7**	**67,5**	***	**29,2**	**54,2**	***	**53,2**	**75,4**	***	**41,6**	**60,1**	***

Sig.: * ≤ 0.05 | ** ≤ 0.01 | *** ≤ 0.001. Blank spaces: Inter-group difference not significant at the 5%-level. The mean value is unweighted, so that every country has the same weight irrespective of population or sample size.

Considering the younger cohort of 2002 and the older cohort of 2018 (the same birth cohorts, (1938–1953), it becomes clear that this cohort had a weak decline in their share of persons in good or very good health. Amongst the low-income tercile, the share declined from 45.6 percent to 41.8 percent, and amongst the high-income tercile from 66.0 to 59.7. In this manner, the high-income tercile lost more absolute percentage points (6.3, with only 3.8 for the low-income group). Although the income position is not necessarily stable and people can leave or enter the three income positions, clearly for those between 1938 and 1953 the health differences between low and high earners did not increase. They rather declined, as these birth cohorts left the middle age (49–64 years) and entered old age (65–80 years). One explanation might be that especially in the low tercile, some with bad health deceased, which improved this group’s share of those in good or very good health.

There are strong correlations between the country values in Table 1: Within 2002 as well as 2018, countries with a high share of respondents in good or very good health in the low-income tercile report high values in the high-income tercile as well (and vice versa). Therefore, good or bad health of income groups are sub-aspects of general good or bad health in the cohort. There seems to be no remarkably trade-off between income groups. Further, a high share in 2002 for a cohort translates into a high share in 2018 for the same cohort (that is, one age group higher). This applies to the low as well as the high tercile. The low (high) tercile in middle age in 2002 is not the same group as the low (high) tercile in high age in 2018, but tendentially, since low- (high) income workers tend to be low- (high) income retirees Pearson’s R-values are between 0.734 and 0.910 (all significant at the 0.1%-level). This means that

(1) within countries, the self-reported state of health of the low tercile is strongly related to the self-reported state of health for the high tercile,
(2) also, within countries, the younger group’s self-reported state of health is strongly related to that of the older group. Put differently, the state of health of the birth cohort of 1938–1953 in 2002 as a middle-aged group (49–64 years) correlates to these cohort’s health status in 2018 as an old age group (65–80 years) (with values of 0.909 for the low-income group in the young group in 2002 and the low-income group in the old cohort in 2018, and 0.880 for the high-income group),
(3) differences between countries are vastly stable, so that countries with a high share of respondents reporting good or very good health in a certain age group in 2002 usually also had high shares in 2018 in the same age group (that is, the later birth cohort).

Better health for the low tercile reduces health inequality. Focusing on absolute inequality and on age groups (not birth cohorts) for the middle age group (49–64 years), improved health of the low tercile is related to lower inequality (Pearson’s R −0.475, not significant at the 5%-level). For the old age group (65–80 years) the correlation is stronger (−0.717, significant at the 0.1%-level). Focusing on the birth cohort of 1938–1953 (middle age in 2002, old age in 2018), the development of the health status of the low tercile of this cohort is weakly negatively related (−0.372, not significant at the 5%-level) to health inequality between this group and the high tercile in 2018. Clearly, such correlations are weakened by the fact that households partly change the income tercile to which they belong.

Further, values for the youngest group in 2002 (age 33–48, birth cohorts 1954–1969) are strongly related to the values of the same birth cohort as it approaches middle age (49–64 years) in 2018 (Pearson’s R. for the low-income tercile 0.830 and the high tercile 0.760).

### 3.2 Development of absolute health inequalities

**Fig. 2:**
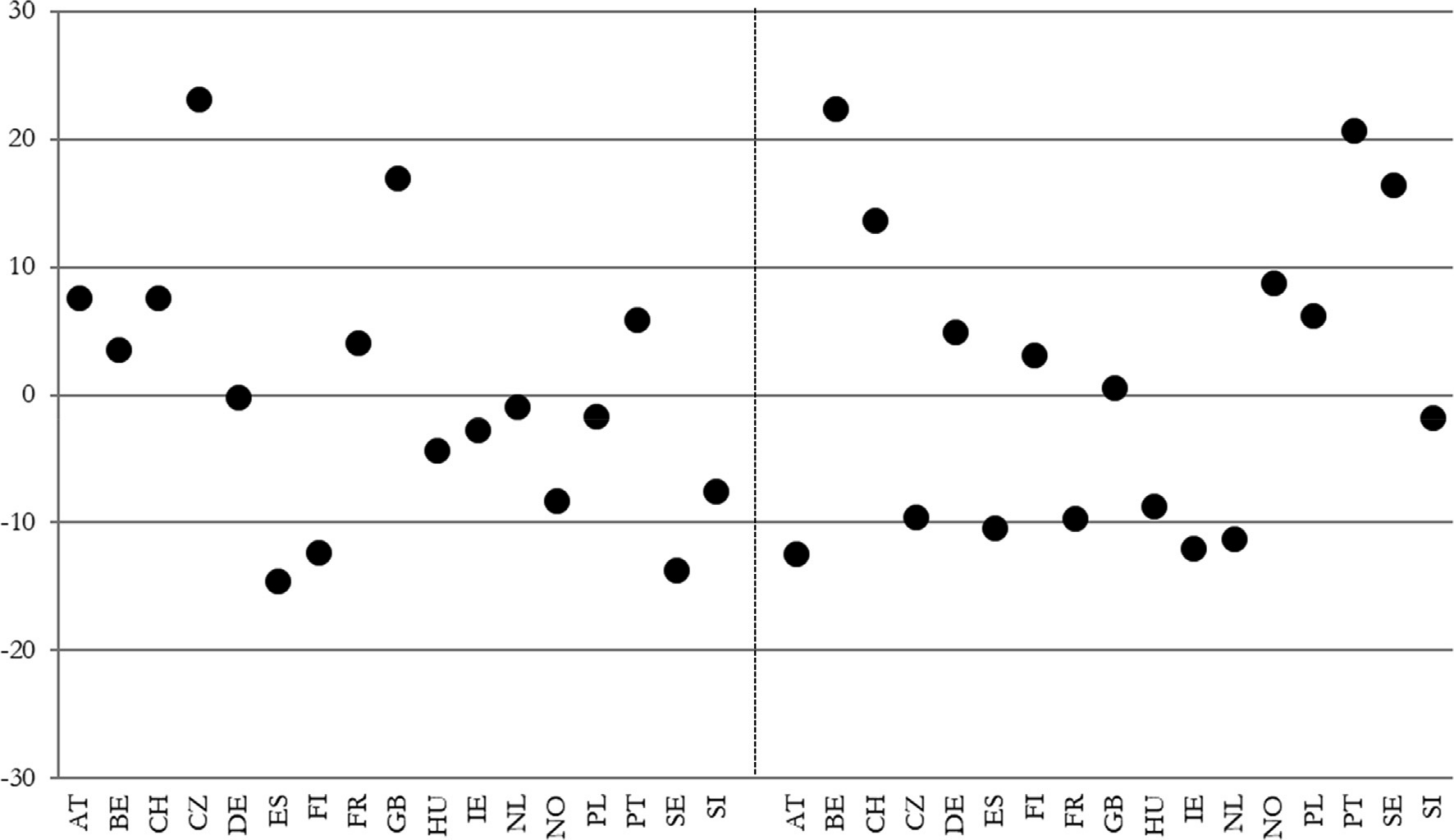
Development of absolute health inequalities between 2002 and 2018 (left side: Age group 49–64, right side: Age group 65–80) (percentage points).

Fig. 2 shows that in the mean across the 17 countries, absolute health inequalities remained vastly unchanged. There is a rise by 0.18 percentage points in the mean for those between 49 and 64 (median: −0.90 percentage points), and a rise by 1.3 percentage points for those between 65 and 80 (median: +0.60 percentage points). Therefore, in contrast to studies mentioned above which refer to time periods before our time span or to time spans overlapping only with the beginning of our time span, we do not even find increasing absolute health inequalities.

### 3.3 Limitations

The ESS question for the “household’s total income, after tax and compulsory deductions, from all sources” (ESS 9 Source Questionnaire) comes with some problems. For example, the intensity of missing responses or answers due to social desirability effects or incorrect / incomplete information on the respondents’ side [9]. Further, considering the effect on health status long-term circumstances and behaviours, the fact that households partly change the income tercile they belong to implies that amongst older age groups respondents may be defined as belonging to a certain income group, although they possibly spent their life vastly in a different income group. This again underlines that we refrain from causal interpretations and merely depict the self-rated health situation. Lastly, it is plausible that respondents assess their own health not solely in comparison to their past or future health, or their assessment of the average health status in their respective society, but partly in comparison to others in their own age group. This may lead to rather positive assessments by the oldest age groups.

## 4 Conclusions

Our study shows a considerable increase of older persons reporting good or very good health between 2002 and 2018, in all four groups examined. Absolute differences between status groups remained stable. In 2018 the high-income tercile of those between 65 and 80 still reported better health than the low-income tercile of those between 49 and 64. Overall, one could argue that the self-rated health has improved in the countries examined. This does not take responsibility off policymakers, as these results need to be investigated further. Our findings, and especially the low shares of those in good or very good health in the lowest tercile, underline the importance of policy measures aiming at increasing the health status of (older) persons, particularly of those with a low-income situation. Health inequalities have social origins and are therefore avoidable [32], at least to some degree. Further, health is affected not only by policies directly focused on health. Yet, the state of research on socio-economic inequality and population health is ambigious. Several older meta studies and some single studies [2], [22] show that higher inequality is related to worse population health, but studies published later rather find no such effects (probably also due to better methods [2], [33]). These results point out that a wider view on (non-spurious) effects from societal circumstances to health and health inequalities could be useful.

## CRediT authorship contribution statement

**Jürgen Bauknecht:** Conceptualization, Software, Data curation, Visualization. **Sebastian Merkel:** Writing – original draft, Visualization, Supervision.

## Declaration of Competing Interest

The authors declare that they have no known competing financial interests or personal relationships that could have appeared to influence the work reported in this paper.
